# Highlights from the 5th Symposium on Biological Data Visualization: Part 1

**DOI:** 10.1186/1471-2105-16-S11-S1

**Published:** 2015-08-13

**Authors:** Jan Aerts, G Elisabeta Marai, Kay Nieselt, Cydney Nielsen, Marc Streit, Daniel Weiskopf

**Affiliations:** 1 Visual Data Analysis Lab, ESAT/STADIUS, KU Leuven, Kasteelpark Arenberg 10, 3001 Leuven, Belgium; 2 iMinds Medical IT, KU Leuven, Kasteelpark Arenberg 10, 3001 Leuven, Belgium; 3 Electronic Visualization Lab, University of Illinois at Chicago, 851 S. Morgan St, Chicago, IL 60607, USA; 4 Center for Bioinformatics, University of Tübingen, Sand 14, 72076 Tübingen, Germany; 5 Department of Pathology and Laboratory Medicine, University of British Columbia and British Columbia Cancer Agency, 675 West 10th Avenue, Vancouver, British Columbia, V5Z 1L3, Canada; 6 Institute of Computer Graphics, Johannes Kepler University Linz, Altenberger Straße 69, 4020, Linz, Austria; 7 VISUS, University of Stuttgart, Allmandring 19, 70569 Stuttgart, Germany

## 

High-throughput and high-resolution experimental methods in biology pose enormous challenges for current biological data visualization approaches. To address these challenges, researchers in the visualization and bioinformatics communities need to engage in the design, implementation, application, and evaluation of novel visualization techniques and tools that provide insight into large and highly complex data sets.

BioVis 2015 - *the fifth Symposium on Biological Data Visualization *- brought together researchers from the visualization, bioinformatics, and biology communities to establish an interdisciplinary dialogue and promote the sharing of expertise between both meeting participants and the communities at large. The meeting educated, inspired, and engaged visualization researchers in problems in biological data visualization as well as bioinformatics and biology researchers in state-of-the-art visualization research. The symposium serves as a platform for researchers from these fields to increase the impact of data visualization approaches in biology. The BioVis 2015 symposium is affiliated with ISMB, the Intelligent Systems for Molecular Biology conference, as a Special Interest Group (SIG) and was colocated with ISMB in Dublin, Ireland, July 10-11 2015.

Each paper was reviewed by researchers from both the bioinformatics and visualization fields and was evaluated for improvements over state-of-the-art and for scientific soundness. The review process was organized in two review cycles. In the first review cycle, each paper was reviewed by three to four reviewers. In the second review cycle, the primary reviewers checked whether the required revisions for conditionally accepted papers were successfully included. Based on the reviewers' scores, reviews, and recommendations, the BioVis 2015 Paper and Publication Chairs and the BMC Bioinformatics Section Editor together selected those that would be published as a BMC Bioinformatics supplement.

The papers from BioVis 2015 appear in two different proceedings: As "Proceedings of the 5th Symposium on Biological Data Visualization: Part 1" in this *BMC Bioinformatics* supplement and as "Proceedings of the 5th Symposium on Biological Data Visualization: Part 2" in *BMC Proceedings* (http://www.biomedcentral.com/bmcproc/supplements/9/S6). From the 21 papers submitted to BioVis 2015, 9 papers are published in this *BMC Bioinformatics* supplement and 5 papers are published in *BMC Proceedings*.

The articles in this supplement cover a wide spectrum of challenging problems in biological data visualization and their solutions. Overall, three main themes arise from the BioVis 2015 articles: *omics, proteins*, and *imaging*. In the *omics *field, Younesy et al. [1] describe *VisRseq*: a user-friendly interface for biologists to use libraries in *R *that provides a method for linking *R*-apps with interactive components. Chelaru et al. [2] expand on the design behind *Epiviz*, another tool for bringing genome visualization and computational environments together. Hennig et al. [3] describe *Pan-Tetris *and Aurisano et al. [4] describe *BactoGeNIE*: both systems are designed for comparing different genomes. The *XCluSim *tool by L'Yi et al. [5] has a more general application field and aims to provide insight into how different clustering results relate to each other. In the *protein *field, Stolte et al. [6] give an overview of the design decisions that underlie *Aquaria*, a visual analytics tool for exploring protein-related data. Finally, three papers are included from the *imaging *field. Topics range from image generation, as discussed by Abdellah et al. [7], to a method for parameter optimization in image processing by Pretorius et al. [9] (e.g. for cell nuclei detection and colour deconvolution for histology), and all the way to graph-based exploration of histology images in the *GRAPHIE *system proposed by Ding et al. [8].

The diversity of topics covered in this issue highlights the wide range of challenges in applying existing visualization techniques to biological data. With this analysis and formalization of our collective experiences, we hope to motivate visualization researchers to think about new problems and new approaches to pressing problems in biology.

## Competing interests

The authors declare that they have no competing interests.
